# Research advances on the pathogenesis and clinical interventions of post-stroke depression

**DOI:** 10.3389/fneur.2026.1789695

**Published:** 2026-03-18

**Authors:** Leqi Gao, Jiazhao Song, Moze Zhao, Ruixin Wang, Yi Chen, Minmin Li, Hongwei An, Zheyi Zhou, Wanyu Tan, Zihao Huang

**Affiliations:** 1 Department of Neurology, Third Affiliated Hospital of Guangxi University of Chinese Medicine/Liuzhou Hospital of Traditional Chinese Medicine, Liuzhou, Guangxi, China; 2 Experimental Tumorpathology, University Hospital Erlangen, Friedrich-Alexander-Universität Erlangen-Nürnberg, Erlangen, Germany; 3 Department of Gastroenterology, The First Affiliated Hospital of Guangxi University of Chinese Medicine, Nanning, Guangxi, China; 4 Department of Preventive Medicine, Affiliated Hospital of Traditional Chinese Medicine, Guangzhou Medical University, Guangzhou, Guangdong, China

**Keywords:** clinical intervention, multidisciplinary management, pathogenesis, post-stroke depression, precision prevention

## Abstract

**Background:**

Post-stroke depression (PSD) is a common neuropsychiatric complication affecting 30–50% of stroke survivors, impairing rehabilitation, quality of life, and prognosis.

**Main body:**

This narrative review synthesizes recent evidence on PSD pathogenesis (neurotransmitter dysregulation, neuroinflammation, impaired neuroplasticity; psychosocial factors such as stress and social support deficits; gene–environment interactions including 5-HTT and BDNF polymorphisms), clinical interventions (pharmacotherapy with SSRIs/SNRIs, psychotherapy including CBT, neuromodulation via rTMS/tDCS/ECT, novel agents such as ketamine, and multidisciplinary models), and prevention (risk stratification, early screening with PHQ-9/HAMD, personalized biological/psychosocial strategies, and digital monitoring).

**Conclusion:**

Despite gaps in long-term data and validated biomarkers, multidisciplinary integrated care and precision medicine approaches offer promising avenues to optimize screening, early intervention, prevention, and long-term outcomes for stroke survivors.

## 1 Introduction

Post-stroke depression (PSD) is a common neuropsychiatric sequela of stroke, characterized by persistent low mood, anhedonia, appetite disturbance, and related depressive symptoms (1). Epidemiological studies estimate that approximately 30–50% of stroke survivors develop PSD (2). PSD is associated with adverse clinical consequences, including poorer engagement in rehabilitation, reduced quality of life, and an increased risk of suicide (3). Despite advances in acute stroke management that have improved survival, PSD remains an important determinant of long-term functional recovery and overall prognosis (4). Accordingly, elucidating the biological and psychosocial mechanisms underlying PSD and establishing effective preventive and therapeutic strategies remain key clinical and research priorities.

Clinically, PSD presents with depressed mood, anhedonia, sleep disturbance, and other affective and somatic symptoms (5). Distinguishing these symptoms from stroke-related deficits (e.g., motor impairment, cognitive dysfunction, or aphasia) is challenging, often resulting in under-recognition or misattribution in clinical practice (6, 7). Evidence suggests that PSD is associated with slower neurological recovery, reduced independence in activities of daily living, impaired social functioning, poorer quality of life, and lower adherence to rehabilitation (8, 9). Therefore, early and accurate identification followed by timely, targeted intervention is essential to improve functional outcomes and overall prognosis (10).

Despite progress in understanding PSD, its pathophysiology is not yet fully understood, and standardized management strategies remain limited. Current research has made notable advances in delineating neurobiological mechanisms, psychosocial determinants, and the relationships between PSD and stroke characteristics or subtypes. For instance, comprehensive reviews by Frank et al. (11) and Guo et al. (12) synthesize robust evidence from clinical cohorts demonstrating consistent associations between elevated pro-inflammatory cytokines (IL-6 and TNF-α) in the acute post-stroke phase and the subsequent incidence of PSD, as corroborated by multiple meta-analyses of acute-phase cohorts (11–13). Lu et al. (14) further elucidate the pivotal role of microglial and astrocytic phenotypic transformation in sustaining neuroinflammatory responses that contribute to depressive symptomatology. However, critical limitations persist, including the scarcity of longitudinal biomarker validation studies and the reliance on observational designs that restrict causal inference. Accumulating evidence from both clinical and preclinical studies implicates neurotransmitter dysregulation, neuroinflammation, and disrupted neuroplasticity as key biological pathways specifically in the pathogenesis of PSD, rather than solely in the acute stroke response (14). While longitudinal cytokine-PSD associations and animal models of post-ischemic depressive-like behavior support potential causal links, full causality in humans remains correlative pending further interventional trials. At the same time, psychosocial factors—such as stress coping styles and the level of family and social support—appear to shape both the onset and course of depressive symptoms (15). Translating these mechanistic insights into scalable interventions remains challenging, necessitating risk-stratified, individualized care supported by emerging evidence. In this review, we synthesize evidence on PSD pathogenesis from neurobiological, psychosocial, and genetic perspectives (16). We also summarize recent advances in pharmacotherapy, psychotherapy, and emerging interventions, intending to support early identification and more precise management of PSD in clinical practice. Improved recognition and targeted treatment may ultimately enhance rehabilitation outcomes and quality of life for stroke survivors.

## 2 Literature search and selection

This narrative review was prepared by searching PubMed, Embase, Web of Science, and Scopus for English-language articles published between January 2018 and December 2025. Primary keywords included “post-stroke depression” OR “PSD” combined with “pathogenesis,” “neuroinflammation,” “intervention,” “prevention,” “rTMS,” “CBT,” etc. Priority was given to meta-analyses of RCTs, large prospective cohorts, longitudinal biomarker studies, and recent clinical guidelines; preclinical and smaller exploratory studies were included selectively to illustrate mechanisms or emerging approaches. This structured yet flexible approach ensures comprehensive coverage while maintaining the narrative character of the review.

### 2.1 Pathogenesis of post-stroke depression

#### 2.1.1 Neurobiological mechanisms

The pathogenesis of PSD remains incompletely understood. Recent evidence indicates that neuroinflammation frequently acts as an upstream driver modulating neurotransmitter imbalance and neuroplasticity deficits. Accordingly, the following discussion organizes the three key processes with neuroinflammation presented first as a central hub, followed by its downstream effects on monoaminergic signaling and neuroplasticity.

Neuroinflammation represents a pivotal mechanism in PSD. Both ischemic and hemorrhagic insults activate central and peripheral immune responses, leading to prolonged release of proinflammatory mediators (17). Elevated cytokines, including tumor necrosis factor-α (TNF-α) and interleukin-6 (IL-6), have been reported after stroke and are associated with depressive symptoms (18). Sustained inflammatory signaling may perturb synaptic transmission and functional connectivity within key emotion-regulation networks, including the prefrontal cortex, hippocampus, and amygdala (19).

This neuroinflammatory cascade subsequently contributes to neurotransmitter dysregulation, a central contributor to PSD. Disruptions in monoaminergic signaling—including serotonin (5-HT), norepinephrine (NE), and dopamine (DA)—are commonly observed post-stroke. In particular, reduced serotonergic tone correlates with symptom severity, while NE and DA alterations affect mood regulation, anxiety, motivation, and attention. These disruptions may stem from cerebral hypoperfusion, neuronal injury, network reorganization, and the upstream effects of sustained inflammation (20).

Impaired neuroplasticity constitutes another important downstream consequence. Stroke can trigger structural and functional alterations in regions supporting affective and cognitive processing, such as the prefrontal cortex and hippocampus (21). Adult neurogenesis occurs primarily in the hippocampal dentate gyrus (not the prefrontal cortex), whereas the prefrontal cortex exhibits dendritic atrophy and reduced synaptic density (22). Hippocampal atrophy, a well-established correlate of depression, reflects reduced neurogenesis and synaptic remodeling and has been linked to impaired stress regulation and emotional control (23). Prefrontal cortex atrophy similarly contributes to depression through (i) impaired top-down regulation of the amygdala, (ii) disrupted dopaminergic projections from the ventral tegmental area, and (iii) HPA-axis dysregulation with elevated cortisol impairing glucocorticoid-receptor feedback (24). Post-injury reorganization may further compromise circuit integrity, increasing vulnerability to depressive symptoms (25).

**Figure 1:**
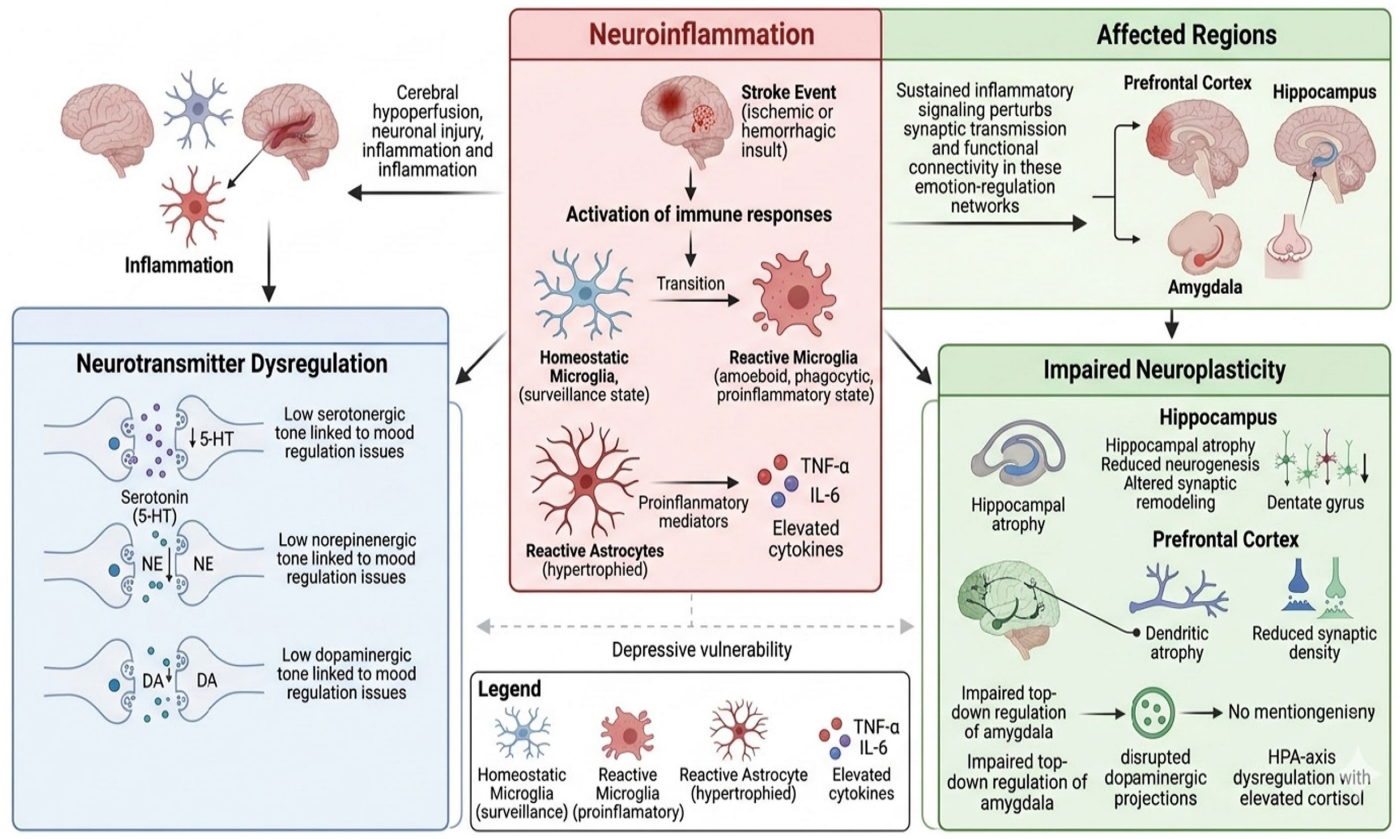
Interconnected neurobiological pathways in post-stroke depression with neuroinflammation as central driver.

Taken together, these interconnected processes—with neuroinflammation acting as a central upstream driver—constitute major neurobiological pathways implicated in PSD. Clarifying how these processes interact may help identify actionable targets and inform more effective, mechanism-based interventions (Figure 1). While these neurobiological mechanisms provide a foundational understanding of PSD pathophysiology, they often interact with psychosocial factors, which can modulate symptom expression and treatment response, as discussed below.

#### 2.1.2 Psychosocial factors

PSD is influenced not only by neurobiological changes but also by psychosocial factors (26). Following stroke, many survivors face functional limitations and psychological distress, which can increase vulnerability to depressive symptoms (27). Studies have associated PSD with psychological stress, reduced social support, coping style, and stressful life events (28).

Psychological stress is a key contributor to PSD. Motor impairment, aphasia, and other stroke-related deficits may disrupt self-identity, reduce autonomy, and force major lifestyle adjustments (29). These challenges often generate anxiety, helplessness, and sustained low mood, which can evolve into depressive symptoms (30). Prolonged stress may also dysregulate stress-responsive systems (e.g., the hypothalamic–pituitary–adrenal axis), with elevated cortisol reported in some patients, potentially amplifying depression risk via downstream neurobiological pathways (31).

Social support is another important determinant. Limited support is linked to greater loneliness and perceived helplessness, both of which increase depression risk. In contrast, emotional and practical support from family and friends can buffer stress, promote emotion regulation, and improve engagement in rehabilitation, which may support recovery (32). Coping strategies and resilience further modulate PSD risk. For instance, adaptive coping (e.g., problem-solving, acceptance, and goal-directed behavior) is associated with fewer depressive symptoms, whereas avoidance and denial correlate with higher risk. Individuals with greater resilience adapt more effectively to post-stroke changes and experience less emotional distress, influenced by personality, prior experiences, and cultural context (33). Finally, stressful life events—such as financial strain, altered family roles, and reduced social participation—can compound psychological burden, especially during early rehabilitation when dependence and uncertainty peak (34).

Finally, stressful life events—such as financial strain, altered family roles, and reduced social participation—can compound psychological burden, especially during early rehabilitation when dependence and uncertainty peak (35, 36). Overall, psychosocial factors—including stress burden, social support, coping style, and life stressors—are closely related to PSD. Effective prevention and management should therefore include assessment of psychological distress, efforts to strengthen social support, and interventions that build adaptive coping skills and resilience.

#### 2.1.3 Genetic and environmental factors

PSD reflects the interplay of neurobiological and psychosocial mechanisms, with growing attention to gene–environment interactions (37).

PSD involves polygenic susceptibility in which multiple genetic variants exert small, additive effects rather than being driven by a single causative gene (20, 38). Specific polymorphisms within key genes—such as the serotonin transporter gene (5-HTT/SLC6A4) in emotion-regulation pathways and the brain-derived neurotrophic factor (BDNF) gene involved in neuroplasticity and neuroprotection—have been linked to increased depressive vulnerability in meta-analyses, although some studies report null findings, highlighting the need for larger cohorts (16, 39). In addition, polymorphisms in inflammatory pathways, including cytokine-related genes such as TNF-α and IL-6, may confer risk by potentiating post-stroke inflammatory responses (40). In many cases, these genetic effects appear to become clinically relevant primarily under unfavorable environmental conditions.

Environmental modulators interacting with genetic susceptibility likewise play a central role in PSD development (41). Stroke itself is a major physiological insult and psychological stressor, and recovery often brings additional pressures, including limited social support, financial strain, and changes in family or occupational roles, all of which can intensify distress and precipitate depressive symptoms (42). Consistently, inadequate family and community support is associated with greater mood instability and higher depression risk (43). Vulnerability may also be shaped by pre-stroke exposures. Adverse childhood experiences—such as exposure to violence or emotional neglect—have been linked to elevated depression risk later in life, potentially through long-lasting effects on neurodevelopment and stress regulation (44). Lower socioeconomic status may further compound risk by increasing chronic stress and constraining access to rehabilitation and mental health resources (45).

In summary, PSD likely arises from the combined effects of genetic predisposition and environmental stressors. Future studies should clarify the nature and timing of gene–environment interactions to enable earlier identification of high-risk individuals and to support personalized prevention and treatment strategies. These neurobiological, psychosocial, and gene–environment pathways interact synergistically, providing the mechanistic foundation for the multimodal interventions reviewed below. These interconnected pathways and corresponding interventions are summarized in Table 1.

**Table 1:** Summary of PSD pathogenesis and clinical interventions.

Domain	Key mechanisms	Interacting factors	Evidence-based interventions	Preventive strategies	Future directions
Neurobiological	- Neurotransmitter dysregulation (e.g., ↓5-HT, NE, DA) (16, 20) - Neuroinflammation (e.g., ↑TNF-α, IL-6) (17–20) - Impaired neuroplasticity (e.g., hippocampal atrophy, ↓neurogenesis) (21–25)	Gene–environment interactions; stroke lesion location (e.g., frontal/basal ganglia) (14, 92)	- SSRIs/SNRIs for monoamine restoration (46, 47) - Anti-inflammatory agents (emerging) (19, 77) - rTMS/tDCS for plasticity enhancement (69, 70)	- Early screening with biomarkers (e.g., cytokine levels) (18, 20) - Risk stratification via neuroimaging (89, 92)	- Precision pharmacogenomics (16, 39) - AI-integrated biomarker panels for circuit-level targeting (112)
Psychosocial	- Psychological stress (e.g., identity loss, helplessness) (29–31) - Social support deficits (32, 43) - Maladaptive coping (e.g., avoidance) (33)	Life events; resilience; cultural/socioeconomic context (34, 36, 45)	- CBT for cognitive restructuring (59–61) -Supportive/group therapy for isolation reduction (62–65) - Family interventions for role adaptation (66)	- Resilience-building programs (33) - Social network assessments (e.g., HADS) (102)	- Digital psychoeducation platforms (95, 98) - Longitudinal resilience tracking via apps (98)
Genetic/environmental	- Polygenic risks (e.g., SLC6A4, BDNF variants) (16, 20, 38, 39) - Environmental stressors (e.g., childhood adversity, socioeconomic strain) (44, 45)	Epigenetic modifications; gut-brain axis influences (77)	- Personalized pharmacotherapy (e.g., genotype-guided SSRIs) (16, 39) - Multidisciplinary care integrating social services (79, 80)	- Gene–environment risk profiling (37) - Community-based support linkages (43, 104)	- GWAS-informed polygenic scores (38) - Microbiome-targeted therapies for gene–environment modulation (77)
Integrated/overarching	Synergistic pathways (e.g., inflammation amplifying neurotransmitter deficits) (13, 17, 20)	Stroke severity; comorbidities (e.g., cognitive impairment) (91, 110)	- Multimodal regimens (e.g., pharmacology +neuromodulation + psychotherapy) (68, 79)	- Digital monitoring (e.g., EMR/remote platforms) (95, 98) - Dedicated longitudinal tracking via integrated EMR and remote platforms for repeated symptom monitoring and adaptive prevention (95, 98) - Individualized plans via multidisciplinary teams (79, 96)	- Randomized trials for efficacy validation (75, 78) - AI-driven adaptive interventions for real-time personalization (112)

### 2.2 Clinical intervention for post-stroke depression

The broad scope of this review—integrating pathogenesis mechanisms (neurotransmitter dysregulation, neuroinflammation, impaired neuroplasticity, psychosocial factors, and gene–environment interactions), a full spectrum of clinical interventions (pharmacotherapy with SSRIs/SNRIs, psychotherapy including CBT, neuromodulation via rTMS/tDCS/ECT, novel agents such as ketamine), prevention strategies, and multidisciplinary integrated care—makes it a single, cohesive, and highly practical resource for a wide clinical audience, including neurologists, psychiatrists, and rehabilitation specialists. While fully maintaining this breadth, usability has been substantially enhanced by reorganizing the intervention content into a clear stepwise clinical workflow (first-line management, escalation for partial responders, management of treatment-resistant PSD, and integrated multidisciplinary care across acute, subacute, and chronic rehabilitation phases), directly supporting rapid reference and bedside application without loss of any original content or mechanistic linkages.

#### 2.2.1 First-line management (pharmacotherapy and psychological interventions)

Pharmacotherapy remains central to first-line management of PSD. Timely pharmacotherapy may improve mood and support functional recovery, with meta-analyses showing SSRIs reduce Hamilton Depression Rating Scale (HAMD) scores by 2–4 points on average (46). Antidepressants—most commonly selective serotonin reuptake inhibitors (SSRIs) and serotonin–norepinephrine reuptake inhibitors (SNRIs) (established first-line therapy; supported by multiple meta-analyses of RCTs, Level Ia evidence)—are the mainstay of care (47). SSRIs are often used as first-line agents due to a generally favorable balance of efficacy and tolerability, while SNRIs may be considered when anxiety symptoms are prominent (48, 49). In stroke survivors, medication choice should be individualized, given frequent comorbidities and polypharmacy, which increases the risks of adverse effects and clinically relevant drug–drug interactions; cardiac safety considerations (e.g., QT-interval effects in susceptible patients) also warrant attention (50, 51). Because antidepressants typically require several weeks for full benefit, close follow-up is important to support adherence and guide dose titration based on response and tolerability (52, 53). Adjunctive agents may be considered in selected cases (e.g., severe agitation or psychotic symptoms) with careful monitoring for sedation, metabolic effects, and potential cerebrovascular risk (54). Although novel approaches such as memantine have been explored, evidence remains preliminary, and further trials are needed (55). Initiation of SSRIs/SNRIs within 1–2 weeks post-stroke in high-risk patients is recommended to optimize early rehabilitation engagement and prevent progression to chronic depression.

Psychological interventions are a key component for improving mood and supporting recovery (56). These approaches can strengthen emotion regulation and resilience and help reduce depressive symptoms (57). Common modalities include cognitive behavioral therapy (CBT), supportive psychotherapy, group-based interventions, and family therapy (58). CBT is among the best-supported treatments for PSD, helping patients identify and modify maladaptive thoughts and behaviors, including negative appraisals of disability and activity avoidance (59). By reframing pessimistic beliefs about recovery and building adaptive coping strategies, CBT can alleviate depressive symptoms and may also improve comorbid anxiety and sleep disturbance (60, 61). Supportive psychotherapy emphasizes empathic listening and emotional validation, fostering self-efficacy and engagement in rehabilitation (62, 63). Group-based programs can reduce isolation, normalize post-stroke emotional responses, and enhance perceived social support, with potential benefits for mood and functioning (64, 65). Family therapy addresses changes in roles and caregiving stress, improving communication and strengthening practical and emotional support, which may facilitate rehabilitation adherence (66). Overall, these interventions complement medication and highlight the need for individualized, needs-based psychological care tailored to post-stroke limitations. These first-line approaches—pharmacotherapy and psychological interventions—draw directly from mechanistic insights into neurotransmitter dysregulation and psychosocial stressors; however, for partial responders, escalation strategies are required to address persistent symptoms, as outlined below.

#### 2.2.2 Escalation strategies for partial responders

If patients show only partial response after 4–6 weeks of optimized first-line therapy, escalation is warranted. Because antidepressants typically require several weeks for full benefit, close follow-up is important to support adherence and guide dose titration based on response and tolerability (52, 53). Adjunctive agents may be considered in selected cases (e.g., severe agitation or psychotic symptoms) with careful monitoring for sedation, metabolic effects, and potential cerebrovascular risk (54). Although novel approaches such as memantine have been explored, evidence remains preliminary, and further trials are needed (55).

Escalation should be considered in the subacute phase (2–12 weeks post-stroke) when initial response is inadequate, including dose optimization, switching from SSRI to SNRI, or augmenting with additional psychological interventions if not already maximized.

#### 2.2.3 Management of treatment-resistant PSD (neuromodulation, ECT, novel agents)

Recent PSD research highlights limitations in conventional therapies, spurring interest in emerging options such as repetitive transcranial magnetic stimulation (rTMS), transcranial direct current stimulation (tDCS), electroconvulsive therapy (ECT), and novel pharmacological strategies (67). These approaches seek to modulate neural circuits through complementary mechanisms, alleviate depressive symptoms, and potentially support neurological recovery (68).

Repetitive transcranial magnetic stimulation and transcranial direct current stimulation (rTMS and tDCS) (promising; moderate-quality RCTs and meta-analyses, Level II evidence, but limited long-term data) are noninvasive neuromodulation techniques that deliver focal magnetic pulses or low-intensity electrical currents to influence cortical networks and promote neuroplasticity (69, 70). Clinical studies suggest that stimulation of the left dorsolateral prefrontal cortex may reduce depressive symptoms and may be particularly relevant for patients who respond poorly to or cannot tolerate antidepressant medications (71, 72). Early evidence suggests that tDCS could facilitate network reorganization and improve emotion regulation, and its favorable safety profile and ease of delivery make it a feasible option during the early recovery phase (73). Combining tDCS with structured cognitive training has been proposed as a way to enhance therapeutic effects.

Electroconvulsive therapy may be considered for severe or treatment-resistant PSD (74). By inducing a controlled generalized seizure under anesthesia, ECT can produce rapid changes in neural activity and neurotransmitter systems, which may translate into faster symptom relief in selected patients (75). However, potential adverse effects—particularly transient cognitive impairment—as well as cardiovascular considerations require careful patient selection, close monitoring, and appropriate risk mitigation. Ketamine, an NMDA receptor antagonist, and anti-inflammatory agents (exploratory; preliminary open-label and small RCTs requiring confirmatory Phase III trials) have demonstrated rapid antidepressant effects in treatment-resistant depression; however, preliminary evidence in PSD remains limited, and well-designed multicenter RCTs are required to confirm efficacy and safety in stroke survivors (76, 77). Taken together, these modalities expand the therapeutic repertoire for PSD and support a more mechanism-informed, multimodal approach. Well-designed multicenter randomized controlled trials are needed to clarify safety, durability of benefit, and how best to integrate these treatments with standard rehabilitation and mental health care (78).

These options are typically reserved for the subacute to chronic phases in patients who fail first-line and escalation strategies.

#### 2.2.4 Integrated multidisciplinary care across rehabilitation phases (acute, subacute, chronic)

PSD is multifactorial, arising from biological, psychological, and social determinants; therefore, single-modality management is often inadequate. Multidisciplinary integrated care provides a patient-centered approach that brings together neurology, psychiatry, rehabilitation medicine, psychology, and nursing to improve assessment and treatment (79). Through coordinated care pathways, this model seeks to relieve depressive symptoms while supporting functional recovery (80).

Close collaboration between neurology and psychiatry is central to integrated management. Neurology leads stroke evaluation and medical treatment, whereas psychiatry provides structured assessment of mood symptoms and oversees psychopharmacological care (81). Joint consultation can facilitate early recognition of high-risk patients and enable individualized plans that combine medication with psychological interventions to prevent symptom worsening. Early, coordinated intervention has been associated with lower PSD incidence and better adherence to rehabilitation in some studies (82).

Integration of rehabilitation services with psychological care is equally important for parallel functional and emotional recovery. Physical, occupational, and speech therapies address motor, cognitive, and communication deficits, while psychological interventions help patients reframe illness-related beliefs, strengthen coping skills, and rebuild self-efficacy (83, 84). Together, these components can enhance participation in rehabilitation and improve quality of life.

Nursing support is indispensable within multidisciplinary models. As frontline providers, nurses can monitor mood changes, provide timely psychosocial support, and deliver health education that promotes engagement and adherence to treatment (85). Structured nursing interventions have been linked to reduced PSD rates and improved rehabilitation outcomes (86).

Family and community resources further extend care beyond the clinical setting. Supportive family involvement may buffer stress and sustain motivation, while community services and social workers can improve continuity of rehabilitation, follow-up, and access to resources (87). These supports enable longer-term management that adapts to patients’ evolving needs.

**Figure 2:**
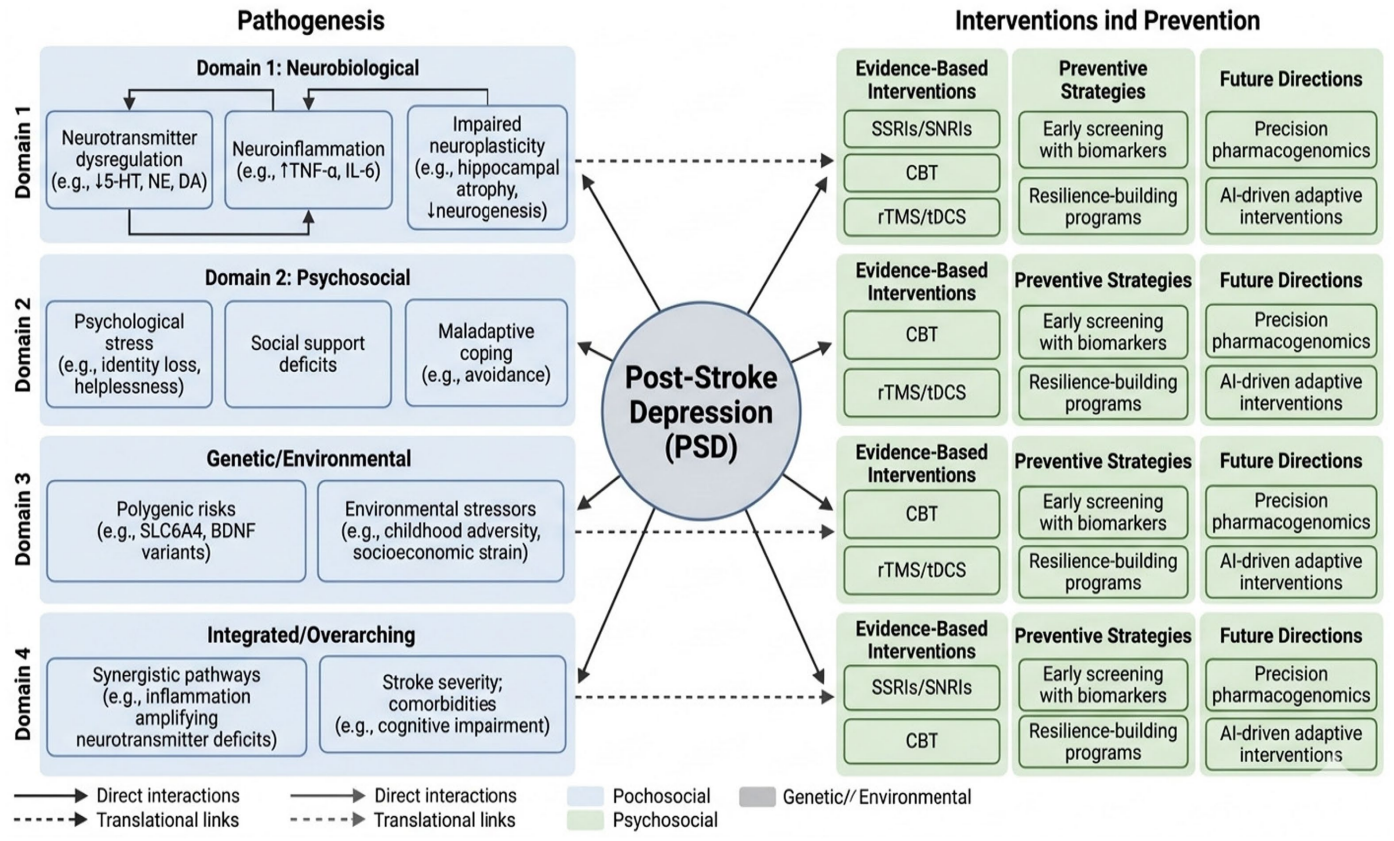
Mechanism diagram of pathogenesis and clinical interventions for post-stroke depression.

Overall, multidisciplinary integrated care offers a practical framework for PSD prevention and treatment by combining cross-disciplinary expertise, systematic assessment, and ongoing adjustment of interventions. Future research should clarify effective models of collaboration and develop standardized care pathways to support comprehensive recovery and durable health benefits in stroke survivors at risk of PSD (88). While the interventions above are grounded in the pathogenic mechanisms outlined in Section 1, many direct translational links remain under active investigation and are highlighted in the Limitations section. The overall mechanisms of PSD pathogenesis and corresponding clinical interventions are summarized diagrammatically in Figure 2.

### 2.3 Prevention of post-stroke depression

#### 2.3.1 Screening and early identification of high-risk patients

Prevention of PSD depends on early recognition and timely intervention in individuals at elevated risk. Because depressive symptoms can be masked by stroke-related neurological deficits, missed or inaccurate diagnoses are common. A structured, evidence-informed approach to screening is therefore critical to reduce PSD burden and improve long-term outcomes (89, 90).

Risk stratification is the foundation of prevention. Studies have identified higher risk among women, older adults, and individuals with a history of depression, more severe post-stroke disability, limited social support, or cognitive dysfunction (91). Lesion-related factors may also contribute; injuries involving the left frontal lobe and basal ganglia have been associated with greater vulnerability to affective disturbance (92). In addition, pain, aphasia, sleep disturbance, and prolonged immobility can further increase the likelihood of depressive symptoms after stroke (93). Early identification should draw on clinical history, neuroimaging findings, functional assessments, and structured psychological evaluation to flag vulnerable patients. Standardized instruments support screening, including the Hamilton Depression Rating Scale (HAMD), Patient Health Questionnaire-9 (PHQ-9), and the Post-Stroke Depression Screening Scale (PSD-S) (94). In patients with cognitive impairment or aphasia, accuracy may be improved by using simplified tools and/or observer-rated measures, supplemented by brief cognitive assessments when appropriate (95).

Multidisciplinary integrated care further strengthens detection and continuity of care, with neurologists, psychiatrists, and psychologists collaborating for integrated neuropsychiatric monitoring (96). Rehabilitation physicians and nursing staff, through frequent patient contact, can identify emerging mood changes, ensure routine reassessment, and coordinate early support (97). Complementing this, digital tools such as electronic medical records (EMR) integrated with remote monitoring platforms and smartphone-based patient-reported outcome apps enable comprehensive longitudinal tracking of mood symptoms (e.g., serial PHQ-9 or HAMD assessments at weekly intervals) alongside rehabilitation progress. This repeated, real-time monitoring throughout the subacute and chronic phases facilitates dynamic risk reassessment, early detection of emerging depressive symptoms, timely adjustment of preventive strategies, and improved adherence, thereby enhancing clinical practicality and long-term outcomes in diverse stroke care settings (98).

Early screening and risk-based identification are central to PSD prevention. Integrating risk stratification with standardized assessment, multidisciplinary collaboration, and technology-supported follow-up can promote earlier intervention and ultimately improve prognosis and quality of life after stroke.

#### 2.3.2 Personalized prevention strategies

Prevention of PSD should reflect patient heterogeneity and adopt individualized strategies aligned with each patient’s biological, psychological, and social context. Stroke survivors differ markedly in lesion characteristics, functional impairment, emotional responses, and available support; therefore, one-size-fits-all prevention approaches are often inadequate. An individualized model requires risk stratification coupled with flexible, multidomain interventions that can be adjusted over time (99).

Clinical and biological factors provide a practical starting point for personalization. Stroke subtype, lesion location, and the severity of neurological injury may influence vulnerability to depression (100). For example, patients with involvement of the left frontal lobe or basal ganglia may be at higher risk of affective disturbance and may benefit from closer monitoring and, when clinically indicated, earlier consideration of pharmacotherapy. In individuals with severe disability or cognitive impairment, rehabilitation planning and psychosocial support should be tailored using neuroimaging, neurological examination, and functional assessments to set realistic goals and improve engagement (101). Personalization should also address psychological status and coping capacity: patients with lower resilience or maladaptive coping may be more prone to depressive symptoms. Standardized tools—such as the Hospital Anxiety and Depression Scale (HADS) and validated measures of perceived social support—can help identify psychological vulnerability and support gaps (102). For those at increased psychosocial risk, early cognitive-behavioral guidance and targeted emotional counseling constitute effective early intervention to strengthen coping skills and facilitate adaptive adjustment (103).

Social and environmental determinants should be incorporated into prevention planning. Patients facing financial strain, limited caregiving capacity, or social isolation may require proactive community follow-up and linkage to social services to build a coordinated family–hospital–community support network (104). Health education should be adapted to cultural context and health literacy to improve understanding, adherence, and motivation for rehabilitation (105). Digital tools can further support ongoing personalization: electronic health records and remote monitoring platforms enable longitudinal tracking of mood symptoms, functional recovery, and adherence, allowing prevention plans to be refined as patients’ needs evolve. In summary, individualized PSD prevention integrates clinical risk, psychological profile, and social context, supported by continuous monitoring and adaptive intervention, with the goal of improving emotional recovery, functional outcomes, and social reintegration after stroke.

## 3 Summary and outlook

Post-stroke depression (PSD) is a frequent and clinically important psychiatric complication during stroke rehabilitation. Its pathogenesis is multifactorial, involving neurobiological processes, psychosocial determinants, and genetic susceptibility (106). This review integrates current evidence on PSD mechanisms and clinical management. We summarize major mechanistic pathways—including neurotransmitter dysregulation, neuroinflammation, and impaired neuroplasticity—and discuss recent progress in pharmacotherapy, psychological interventions, emerging neuromodulation strategies, novel pharmacological approaches, and multidisciplinary models of care. We also address prevention, outlining screening approaches for high-risk groups and personalized strategies aimed at earlier detection and more precise management.

Clinical practice is increasingly moving beyond single-modality treatment toward integrated, multidomain care (107). While pharmacotherapy remains a central option for symptom control, responses vary, and treatment may be constrained by adverse effects and drug–drug interactions. As a result, psychological interventions, rehabilitation-based programs, and structured social support are being incorporated more consistently to promote both emotional well-being and functional recovery (108). At the same time, neurostimulation techniques (e.g., rTMS and tDCS) and emerging medications may broaden options for patients who do not respond adequately to standard treatment (109). Multidisciplinary collaborative care models further enhance continuity and coordination across the rehabilitation trajectory. Despite developments, gaps persist, including limited high-quality evidence on long-term effectiveness and undefined care pathways tailored to stroke subtype (110). Predictive biomarkers, such as BDNF polymorphisms, remain exploratory and require validation for routine use (111).

## 4 Limitations of current evidence

Although this review synthesizes recent advances, several limitations of the current PSD literature must be acknowledged. Substantial heterogeneity exists in PSD diagnostic criteria and assessment tools across studies, limiting direct comparability. Most intervention trials are short-term (≤6 months follow-up), with sparse data on sustained efficacy and safety beyond one year. Many neuromodulation and novel-agent studies (e.g., rTMS, tDCS, ketamine) involve relatively small samples and are conducted predominantly in Asian cohorts, reducing generalizability to hemorrhagic stroke and Western/non-Asian populations. Mechanistic associations, particularly cytokine-PSD links, gene–environment interactions, and neuroimaging correlates, remain largely correlative rather than fully causal, pending confirmation through well-designed interventional trials. Evidence levels across interventions are now explicitly graded as established (Level Ia, e.g., SSRIs/SNRIs), promising but limited (Level II, e.g., rTMS/tDCS), or exploratory (e.g., ketamine and anti-inflammatory agents requiring confirmatory Phase III trials) to avoid overstatement. These limitations underscore the urgent need for larger, longer-term, multicenter, and more diverse studies with standardized outcomes and validated biomarkers.

Future studies will need to adopt a precision-oriented approach, integrating genomics, neuroimaging, and multimodal clinical data to clarify molecular and circuit-level mechanisms of PSD (112). These mechanistic insights are now being directly translated into precision-medicine pathways, such as genotype-guided SSRI selection (5-HTT/BDNF polymorphisms), inflammation-targeted adjunctive therapies, and circuit-specific neuromodulation (rTMS/tDCS over prefrontal-hippocampal networks), enabling truly personalized care from the acute phase through chronic rehabilitation. In parallel, digital health tools and artificial intelligence may support longitudinal monitoring, earlier risk identification, and iterative adjustment of patient-specific interventions. Together with stronger evidence from well-designed trials and effective multidisciplinary collaboration, these advances could help build a continuous care pathway from the acute phase through rehabilitation, ultimately improving quality of life and long-term outcomes for stroke survivors.
